# Opinion: Application of extracorporeal shock wave therapy in nervous system diseases

**DOI:** 10.3389/fneur.2023.1281684

**Published:** 2023-12-19

**Authors:** Leon L. J. Jokinen, Tobias Wuerfel, Christoph Schmitz

**Affiliations:** Department of Neuroanatomy, Faculty of Medicine, Ludwig-Maximilians University of Munich, Munich, Germany

**Keywords:** extracorporeal shock wave therapy, focused extracorporeal shock waves, radial extracorporeal shock waves, ESWT, rESWT, fESWT

## 1 Introduction

We became aware of the article “Application of extracorporeal shock wave therapy (ESWT) in nervous system diseases: A review” by Guo et al. (1) published in Frontiers in Neurology. Unfortunately the information provided in the Section “Principles of ESWT” is partly incorrect and misleading. The first author of this commentary (LJ) suffers from an extreme disability (tetraplegia from C4), is regularly treated with radial ESWT because of his spasticity, has not needed any related medication since then, particularly no injection of BTX-A, and therefore has a special interest in correct reporting about ESWT in the literature. All authors are actively involved in ESWT research (2–5).

**Both focused extracorporeal shock waves (fESWs) and radial extracorporeal shock waves (rESWs) used in ESWT have phases of positive and negative pressure and can generate cavitation**.

**Figure 1:**
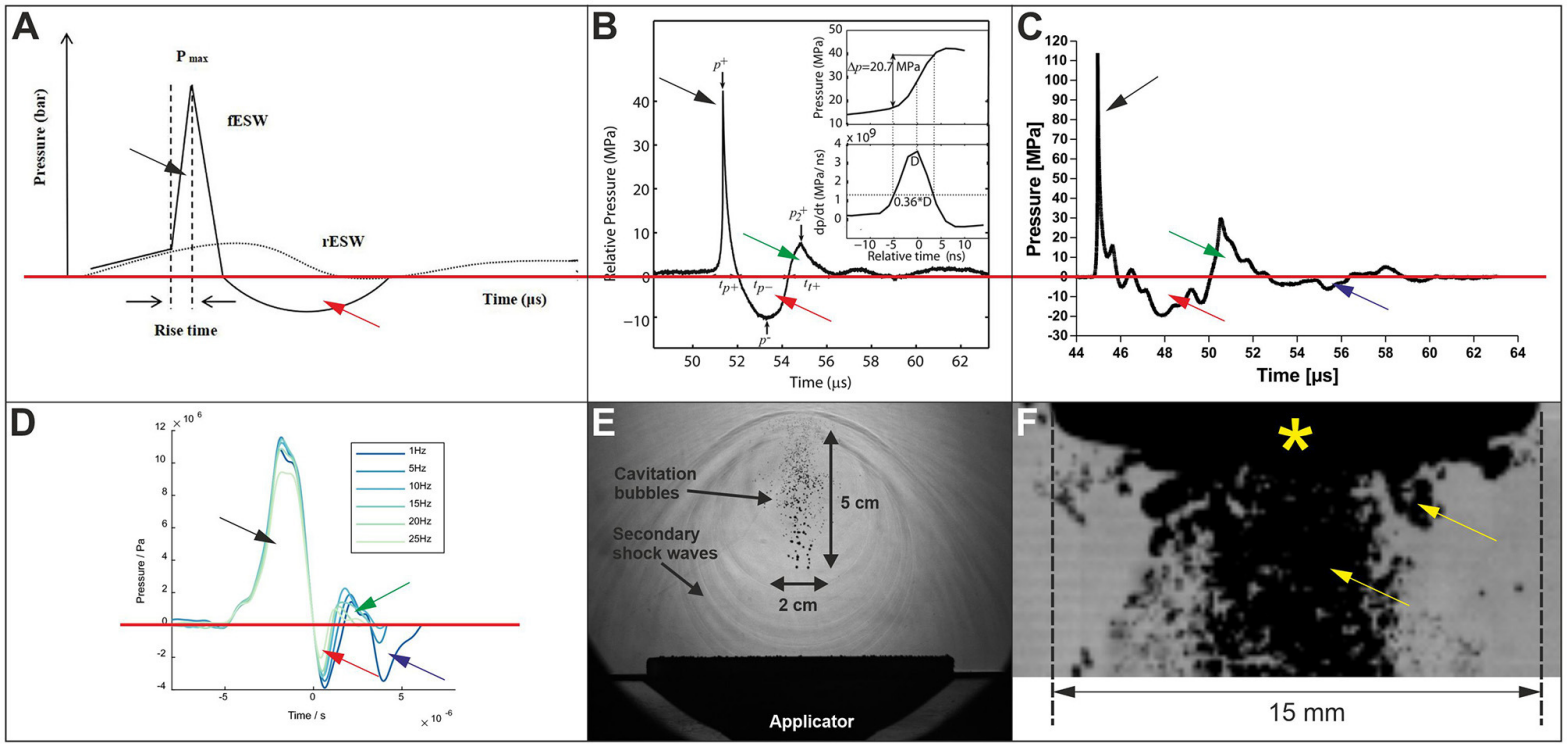
Waveform characteristics of focused and radial extracorporeal shock waves as illustrated in (1) **(A)** and published in (3, 6, 7) **(B–D)**, as well as cavitation fields (yellow arrows) generated by focused **(E)** and radial **(F)** extracorporeal shock waves (4, 5). Details are in the text. The yellow asterisk in **(F)** indicates the applicator of the handpiece of the rESWT device used. **(A)** was taken from (1) published under the CC-BY license; **(B)** was taken from (6) with permission from the publisher^1^; and **(C–F)** were taken from (3)/(7)/(4)/(5) published under the CC-BY license (3), the CC BY 2.0 license (4), or the CC BY 4.0 license (5, 7), respectively. The publications (3–5) are from the senior author of this commentary (CS).

Figure 1A of this commentary shows the waveforms of fESWs and rESWs provided in Figure 1 in Guo et al. (1); Figures 1B, C show real waveforms of fESWs published in the literature (3, 6); and Figure 1D shows real waveforms of rESWs published in the literature (7). These published waveforms differ from the illustrations in Guo et al. (1). Specifically, after an initial phase of positive pressure (black arrows in Figures 1A–D) followed by a necessary phase of negative pressure (red arrows in Figures 1A–D; note that a pressure wave absent a tensile phase is not possible) both fESWs and rESWs can show a second phase of positive pressure (green arrows in Figures 1B–D) followed by a second phase of negative pressure (blue arrows in Figures 1C, D). Of note, the article by Guo et al. (1) is not the only one that shows incorrect representations of the waveforms of fESWs and rESWs. An equally incorrect representation of rESWs (pressure wave absent a tensile phase) can also be found, for example, in Figure 26.2 in Zwerver et al. (8).

Of particular note, both fESWs and rESWs can generate cavitation as a consequence of the negative pressure. This is shown for fESWs in Figure 1E taken from (4) and for rESWs in Figure 1F taken from (5); both publications (4, 5) are from the senior author of this commentary (CS). In contrast, the illustration of the waveform of rESWs provided by Guo et al. (1) (Figure 1A) does not show any negative pressure, which would prevent the formation of cavitation bubbles.

Furthermore, according to the International Standard IEC 61846 (9) the rise time of shock waves is defined at the focus as the time taken for the instantaneous acoustic pressure to increase from 10 to 90% of the peak-positive acoustic pressure, which is different from the illustration provided by Guo et al. (1) in which the rise time is indicated as the time taken for the instantaneous acoustic pressure to increase from approximately 14–100% of the peak-positive acoustic pressure (Figure 1A).

Moreover, extracorporeal shock waves are not characterized by symmetry and a slow, disproportionally large, pressure rise before the onset of the shock front as suggested by the presentation in Guo et al. (1) (Figure 1A). Rather Figures 1B–D show the correct, asymmetrical waveforms of fESWs and rESWs from real measurements according to IEC 61846 (9).

**Some fESWT devices used in contemporary ESWT generate true shock waves, whereas others do not**.

Guo et al. (1) stated that “as an acoustic wave, fESW is characterized by its high pressure of more than 1,000 bar (100 MPa), an extremely short rise time (<10 ns), a short duration (<10 ms), and a broad frequency spectrum (16–20 MHz)”. This description of fESWs is very similar to an earlier description provided by Ogden et al. (10): “A shock wave is a sonic pulse that has certain physical characteristics. There is a high peak pressure, sometimes more than 100 MPa (500 bar), but more often approximately 50–80 MPa, a fast initial rise in pressure during a period of <10 ns, a low tensile amplitude (up to 10 MPa), a short life cycle of ~10 μs, and a broad frequency spectrum, typically in the range of 16 Hz to 20 MHz.” Of note, in Ogden et al. (10) the life cycle of fESWs was correctly given as ~10 μs, which is in line with the waveforms shown in Figures 1B, C, whereas Guo et al. (1) defined the duration of fESWs <10 ms which is 1,000 times longer.

Furthermore, Ogden et al. (10) provided the frequency range of fESWs correctly as 16 Hz to 20 MHz, whereas the frequency range provided by Guo et al. (1) of 16–20 MHz is not correct.

The waveform shown in Figure 1C of the piezoelectric fESWT applicator F10G4 (Richard Wolf, Knittlingen, Germany) operated at highest machine settings is in line with the definition provided by Ogden et al. (10), whereas the waveform shown in Figure 1B of the electromagnetic fESWT device Duolith SD1 (Storz Medical, Tägerwillen, Switzerland) operated at highest machine settings is not. Figure 1C in Guo et al. (1) shows the focused handpiece of the Duolith SD1 device (Storz Medical). Waveform characteristics of the fESWs generated by the Duolith SD1 device (Storz Medical) were reported in 2007 in (6) as follows: maximum pressure 42.7 MPa, rise time 8–500 ns, no formation of true shock waves for any machine settings.

**Like fESWs, rESWs can possess non-linearity**.

Guo et al. (1) stated that “unlike fESW, radial extracorporeal shock wave (rESW) does not possess the shock wave characteristics of a short rise time, a high peak pressure, and non-linearity”. In contrast to this description, Cleveland et al. (11) demonstrated already in 2007 non-linear distortion of the rESWs generated using the rESWT device Swiss DolorClast (Electro Medical Systems, Nyon, Switzerland) (Figure 1D). However, the non-linear distortion was not strong enough to result in a shock front. Of note, this is different from the description by Guo et al. (1) that rESWs do not possess non-linearity. Furthermore, Cleveland et al. (11) reported a rise time of 800 ns of the rESWs generated by the Swiss DolorClast (Electro Medical Systems), which is not too different from the 500 ns reported in (6) for the Duolith SD1 (Storz Medical) at low machine settings.

**A scientifically correct classification of shock waves used in contemporary ESWT would have to distinguish between focused shock waves, focused pressure waves and radial pressure waves**.

Guo et al. (1) stated that “some scholars even call ‘rESW' ‘radial pressure waves' because rESW uses the energy generated from compressed gas to drive the bullet body to the treated tissue area in a pulsed manner”. In this regard it is of note that Cleveland et al. (11) reported that piezoelectric and electromagnetic fESWT sources (such as the F10G4 device from Richard Wolf and the Duolith SD1 device from Storz Medical) use focusing but do not generate shock waves at the source. Rather, they rely on non-linear propagation distortion to produce a shock along the path to the focus. For mid and high-amplitude settings, the waveforms are shocked and the peak amplitudes and rise times are comparable to those of electrohydraulic sources (as shown in Figure 1C for the F10G4 device from Richard Wolf). However, at low-amplitude settings the waveforms do not contain shocks (11), as demonstrated by Perez et al. (6) for the Duolith SD1 (Storz Medical) at any machine settings (Figure 1B). Therefore, it would be correct to distinguish between true focused shock waves (Figure 1C), focused pressure waves (Figure 1B) and radial pressure waves (Figure 1D). However, for several good reasons, this is not done in the literature. One of these reasons is that differences in molecular and cellular mechanisms of action between true focused shock waves and focused pressure waves were not reported in the literature.

## 2 Discussion

It is beyond the scope of this commentary to discuss to what extent the incorrect and misleading description of the principles of ESWT in the article by Guo et al. (1) had influence on the other sections of their article. In our opinion the readers of Frontiers in Neurology should be informed that the principles of ESWT are different than outlined in the article by Guo et al. (1), and the molecular and cellular mechanisms of action of fESWs and rESWs on nervous tissue presented by Guo et al. (1) are incomplete. Our recent publication (2) provides a comprehensive review of the molecular and cellular mechanisms of action of fESWs and rESWs on nervous tissue, and Figures 1, 2 in (12) [also published by the senior author of this commentary (CS)] provide an overview on the physical mechanisms of generating fESWs and rESWs.

## Author contributions

LJ: Conceptualization, Formal analysis, Investigation, Validation, Writing – review & editing. TW: Conceptualization, Formal analysis, Investigation, Validation, Writing – review & editing. CS: Conceptualization, Data curation, Formal analysis, Investigation, Methodology, Project administration, Resources, Supervision, Validation, Visualization, Writing – original draft.

## Footnotes

^1^ https://s100.copyright.com/CustomerAdmin/PLF.jsp?ref=8378be67-f58e-41e8-9f75-39f33cf202d0
